# Complexity of Wake Electroencephalography Correlates With Slow Wave Activity After Sleep Onset

**DOI:** 10.3389/fnins.2018.00809

**Published:** 2018-11-13

**Authors:** Fengzhen Hou, Zhinan Yu, Chung-Kang Peng, Albert Yang, Chunyong Wu, Yan Ma

**Affiliations:** ^1^Key Laboratory of Biomedical Functional Materials, School of Science, China Pharmaceutical University, Nanjing, China; ^2^Division of Interdisciplinary Medicine and Biotechnology, Department of Medicine, Beth Israel Deaconess Medical Center/Harvard Medical School, Boston, MA, United States; ^3^Key Laboratory of Drug Quality Control and Pharmacovigilance, Ministry of Education, China Pharmaceutical University, Nanjing, China; ^4^Department of Pharmaceutical Analysis, China Pharmaceutical University, Nanjing, China

**Keywords:** EEG, brain activity, non-linear, sleep medicine, sleeps stages, complexity

## Abstract

Sleep electroencephalography (EEG) provides an opportunity to study sleep scientifically, whose chaotic, dynamic, complex, and dissipative nature implies that non-linear approaches could uncover some mechanism of sleep. Based on well-established complexity theories, one hypothesis in sleep medicine is that lower complexity of brain waves at pre-sleep state can facilitate sleep initiation and further improve sleep quality. However, this has never been studied with solid data. In this study, EEG collected from healthy subjects was used to investigate the association between pre-sleep EEG complexity and sleep quality. Multiscale entropy analysis (MSE) was applied to pre-sleep EEG signals recorded immediately after light-off (while subjects were awake) for measuring the complexities of brain dynamics by a proposed index, CI_1−30_. Slow wave activity (SWA) in sleep, which is commonly used as an indicator of sleep depth or sleep intensity, was quantified based on two methods, traditional Fast Fourier transform (FFT) and ensemble empirical mode decomposition (EEMD). The associations between wake EEG complexity, sleep latency, and SWA in sleep were evaluated. Our results demonstrated that lower complexity before sleep onset is associated with decreased sleep latency, indicating a potential facilitating role of reduced pre-sleep complexity in the wake-sleep transition. In addition, the proposed EEMD-based method revealed an association between wake complexity and quantified SWA in the beginning of sleep (90 min after sleep onset). Complexity metric could thus be considered as a potential indicator for sleep interventions, and further studies are encouraged to examine the application of EEG complexity before sleep onset in populations with difficulty in sleep initiation. Further studies may also examine the mechanisms of the causal relationships between pre-sleep brain complexity and SWA, or conduct comparisons between normal and pathological conditions.

## Introduction

Sleep medicine has been increasingly recognized as an important discipline in recent decades; however, the current limitations of electroencephalography (EEG)-based sleep analysis and quantification may have led to ongoing controversy. Sleep is a complex physiological process that involves functions of every organ system at different levels. Alternative metrics have been proposed, aiming to provide insights into the dynamics of sleep. In sleep medicine, EEG is one of the most frequently recorded biological signals, but it is mainly used as the basis for scoring sleep stages in sleep laboratories and clinics, as sleep is classified as either rapid-eye-movement (REM) sleep or non-REM (NREM) sleep (including sleep stages N1, N2, and N3). Owing to the non-linear and dynamic features of EEG, non-linear approaches may lead to better understanding of the profound complexity of sleep.

Non-linear dynamics theory provides new opportunities for understanding the behavior of EEG (Acharya et al., 2005). Previously, EEG has been used to mark features of sleep (He et al., 2005; Janjarasjitt et al., 2008; Yeh et al., 2013; Abeysuriya et al., 2014), and it has been reported that nonlinearity depends on sleep stage (Shen et al., 2003). Recently, studies have increasingly used non-linear methods to investigate the nature of brain activities during sleep (Ma et al., 2018), but there are still limitations in the existing literature. The full advantages of non-linear approaches have yet to be determined (Ma et al., 2018).

According to the complexity theories, somewhat higher complexity is associated with relatively improved health conditions and greater chances of survival (Costa et al., 2002a, 2005), while a reduction in or loss of complexity is often associated with imbalance or disturbed physiological conditions, usually implying disease or aging (Goldberger et al., 2002). In many studies, complexity-based metrics of the dynamics of a physiological system have demonstrated better prognostic power (Mejaddam et al., 2013; Lin et al., 2014; Vandendriessche et al., 2014; Moshirvaziri et al., 2016; Chiu et al., 2017; Ma et al., 2017). The complexity theories also suggest that different levels of complexity can indicate whether a system is under stress or relatively relaxed (Costa et al., 2002a, 2005; Goldberger et al., 2002). In a novel application of complexity theory, Casali et al. found that measuring complexity can provide a reliable way to discriminate the level of consciousness in single individuals during wakefulness, sleep, and anesthesia, as well as in patients who had emerged from coma and recovered a minimal level of consciousness (Casali et al., 2013). When complexity is used in the evaluation of sleep, studies have shown that complexity indices (e.g., entropy) decrease as sleep gets deeper, and reach their lowest level when slow wave sleep (SWS) occurs (Ma et al., 2018). Moreover, Abásolo et al. found that activated brain states—wakefulness and REM sleep—are characterized by higher complexity compared with NREM sleep (Abásolo et al., 2015). Based on these well-established theories and previous studies, investigating the sleep-related temporal structure of brain activity based on measures such as multi-scale entropy (MSE) (Costa et al., 2002a, 2005), the perturbational complexity index (Casali et al., 2013), or Lempel-Ziv complexity (Abásolo et al., 2015) should provide insights that go beyond those obtained with conventional techniques for signal analysis.

In sleep medicine, one question that remains unsolved is whether the complexity of brain waves in the pre-sleep state or during sleep latency can determine or predict sleep quality. In fact, most people with insomnia complain of being unable to fall asleep because they cannot switch off their “racing” mind (Lichstein and Rosenthal, 1980; Espie et al., 1989; Harvey, 2000). Under high mental load, the sense of urgency about falling asleep adversely affects sleep onset latency (Ansfield et al., 1996). Since stressful brain activities always show higher complexity, we can reasonably hypothesize that lower complexity of brain waves at pre-sleep status can facilitate sleep initiation, reduce sleep latency, and further lead to a high quality of sleep characterized by sufficient deep sleep or SWS.

Slow wave sleep SWS is defined as the state in which large-amplitude, low-frequency waves are dominant and it occurs when delta rhythm is dominant in the EEG signal. Slow wave activity (SWA), which is equivalent to delta activity and encompasses components of the EEG signal in the frequency range of ~0.5–4.5 Hz, is considered to be one of the most important functional EEG parameters during sleep (Brunner et al., 1990; Peter Achermann and Borbély, 2011). Under physiological conditions, SWA is commonly used as a quantitative measure of NREM sleep dynamics and an indicator of sleep depth or sleep intensity (Borbély and Achermann, 1999); Olivier et al. (2010). Fast Fourier transform (FFT) analysis is the most popular method for quantifying SWA. However, it has intrinsic limitations in capturing the underlying dynamics of brain oscillations (Ma et al., 2018). First, FFT analysis takes complex EEG oscillations, composed of sine waves with different frequencies (Campbell, 2009), and decomposes them into frequency component bands, such as beta, alpha, theta, and delta. However, it has long been known that brain oscillation is not a linear combination of these arbitrary frequency components, a property called “nonlinearity” (Bedard et al., 2006). Second, FFT analysis assumes that none of these frequency components changes in amplitude or shape as time evolves, which is clearly against what has been observed in complex brain oscillations, a property called “nonstationarity” (Campbell, 2009). In recent years, ensemble empirical mode decomposition (EEMD) has been adopted to solve this problem (Wu and Huang, 2005). EEMD is an adaptive and noise-assisted data analysis method that is based on local characteristics of the data, requiring no predefined basis. EEMD decomposes an original non-linear and non-stationary signal into a series of simple intrinsic mode functions (IMFs), and has the advantage that every IMF can be physically meaningful via the quantification of the instantaneous amplitude and frequency (Wang et al., 2012). EEMD has become popular for analyzing EEG signals in recent years (Chen et al., 2010, 2014; Kuo et al., 2011; Bizopoulos et al., 2013; Al-Subari et al., 2015a,b; Kanoga and Mitsukura, 2015; Bai et al., 2016; Zeng et al., 2016; Gotz et al., 2017; Hassan and Bhuiyan, 2017). Employing EEMD to quantify SWA might lead to additional findings.

Therefore, the aim of the present study was to examine whether the complexity of brain waves in the pre-sleep state is associated with sleep quality. We hypothesized that lower complexity of brain waves before sleep may play a potential facilitating role in wake-sleep transition and improve the subsequent sleep depth. To this end, the MSE method was applied to EEG signals recorded during the first 5 min after light-off, when the participants were still awake. Sleep latency was determined by manual scoring, and sleep depth was quantified by SWA using an EEMD-based approach or traditional FFT analysis.

## Methods

### Overnight polysomnography (PSG)

Overnight polysomnography (PSG) data obtained from the Sleep Heart Health Study-1 (SHHS-1) were used in this study. The SHHS was a multi-center cohort study implemented by the National Heart Lung and Blood Institute to determine the cardiovascular and other consequences of sleep-disordered breathing, and its characteristics have been described in detail elsewhere (Quan et al., 1997; Redline et al., 1998). Unattended overnight PSG was performed with a portable PS-2 system (Compumedics, Abottsville, Australia). Sensors were placed and equipment was calibrated during an evening home visit by a certified technician. Data collection included C3/A2 and C4/A1 EEGs, sampled at 125 Hz; right and left electrooculograms; a bipolar submental electromyogram; thoracic and abdominal excursions (inductive plethysmography bands); airflow (detected by a nasal-oral thermocouple (Protec, Woodinville, WA); finger pulse oximetry (Nonin, Minneapolis, MN) sampled at 1 Hz; electrocardiogram sampled at 125 Hz; body position (mercury gauge sensor); and ambient light (on/off, by a light sensor secured to the recording garment). After equipment retrieval, the data were forwarded to a central reading center (Case Western Reserve University, Cleveland, OH) for scoring according to a standard protocol. Finally, every 30 s epoch was scored (Thomas et al., 2007). The polysomnographic methods, scoring protocol, and quality assurance procedures were as previously described (Quan et al., 1997; Redline et al., 1998; Thomas et al., 2007).

Sleep variables derived from visual scoring were calculated for each participant, including: total sleep time (TST) (time spent asleep between sleep onset and light-on); wake time after sleep onset (WASO, total amount of time awake after falling asleep); and the duration of each sleep stage, calculated as a percentage of TST. Sleep latency, defined as the period from light-off to the first three consecutive epochs of stage N1 sleep or an epoch of any other stage, was also computed.

### Subjects

The study included 103 healthy subjects who met the inclusion criteria: (1) no usual daily alcohol intake; (2) no benzodiazepines or non-tricyclic antidepressants intake within 2 weeks of the SHHS-1 visit; (3) no history of diabetes; (4) no history of stroke; (5) no hypertension status based on second and third blood pressure readings or current treatment with anti-hypertensives; (6) no self-reported hypertension; (7) no self-reported sinus trouble; (8) no coronary angioplasty, heart failure, heart attack, pace maker, or stroke; (9) apnea–hypopnea index, representing the number of apnea and hypopnea events with ≥3% oxygen desaturation per hour of sleep, of < 5; (10) the entire recording was scored, and scoring started before light-off and ended after light-on; (11) no more than 30 min of the sleep period had either lost or unscorable EEG, respiratory, or oximetry data; (12) the time spent on sleep was no less than 50% of the total time spent in bed; (13) at least one epoch during each sleep stage, i.e., REM, N1, N2, and N3; (14) sleep latency no less than 5 min. See the Supplementary Material for identifiers of the included subjects, which were created by the National Sleep Research Resource team for easier matching with file downloads.

### The theory of multiscale entropy (MSE)

MSE was introduced by Costa et al. (2002a,b); Costa et al. (2005) to quantify the complexity of biologic systems. Entropy-based methods characterize uncertainty about a source of information and the probability distribution of the samples drawn from it. The entropy increases with the degree of disorder and reaches its maximum in completely random systems. However, an increase in the entropy may not always be associated with an increase in dynamical complexity. For instance, a randomized time series has higher entropy than the original time series, although the process of generating surrogate data destroys correlations and degrades the information content of the original signal. This inconsistency may be related to the fact that widely used entropy measures are based on single-scale analysis and do not take into account complex temporal fluctuations. Therefore, MSE has been proposed as a method for assessing complexity by measuring the entropy inherent in a time series over multiple time scales.

The procedures involved in calculating MSE have been well reviewed (Costa et al., 2002a,b, 2005) and can be summarized in the following three steps (Yang et al., 2013): (1) construction of a coarse-grained time series according to a scale factor; (2) quantification of the sample entropy of each coarse-grained time series; and (3) examination of the sample entropy profile over a range of scales. The length of each coarse-grained time series is equal to the length of the original time series divided by the scale factor. For scale 1, the time series is simply the original time series. Sample entropy is defined by the negative natural logarithm of the conditional probability that a dataset of length *N*, having repeated itself within a tolerance *r* (similarity factor) for *m* points (pattern length), also repeats itself for *m* + 1 points, without allowing self-matches (Richman and Moorman, 2000).

One requirement in the calculation of sample entropy is to determine the pattern length *m* and similarity factor *r*. In this study, we set *m* to 2 and *r* to 0.15 × SD, where SD is the standard deviation of the analyzed time series. As 30 scales were considered and the entropy on each scale does not necessarily have a specific physiological meaning, a complexity index, called CI_1−30_, was additionally employed in the current study. CI_1−30_ was defined as the mean value of the entropies from scale 1 to scale 30, as in many other published studies (Vieira et al., 2017). CI_1−30_ provides insight into the integrated complexity of the system over the time scales of interest. See the Supplementary Material for the MATLAB code for MSE analysis.

### The theory of EMD and EEMD

Empirical mode decomposition (EMD) is an adaptive data analysis method based on local characteristics of the data, requiring no predefined basis. The details of the method can be found in the work of Huang (2000).

In the EMD approach, the targeted data *x*(*t*) is decomposed in terms of IMFs, *c*_*j*_, i.e.,

(1) $$\begin{array}{r} x\left(t\right)=\sum_{j=1}^{n}c_{j}+r_{n} \end{array}$$

where *r*_*n*_ is the residue of data *x*(*t*) after *n* number of IMFs have been extracted. IMFs are simple oscillatory functions with varying amplitude and frequency. In practice, the EMD is implemented through a sifting process that uses only local extremes, and the process stops when the residue, *r*_*n*_, becomes a monotonic function from which no more IMFs can be extracted (Huang, 2000).

However, mode-mixing, defined as any IMF consisting of oscillations of dramatically disparate scale, can be caused by intermittency of the driving mechanisms and obstructs the true physical interpretations (Wu and Huang, 2005). Therefore, EEMD was developed to alleviate this drawback, making use of the facts that adding noise to the data can provide a uniformly distributed reference scale, and the means of the corresponding IMFs of different white noise series are likely to cancel each other out. The detailed steps for EEMD are (Wu and Huang, 2005): (1) add a white noise series to the targeted data; (2) decompose the data with added white noise into IMFs based on EMD; (3) repeat step (1) and step (2), but with different white noise series each time; and (4) obtain the (ensemble) means of corresponding IMFs of the decompositions as the final result. The MATLAB code for EEMD was shared by RCADA (http://rcada.ncu.edu.tw/research1.htm).

A Hilbert transform can then be used to calculate the instantaneous frequency of the IMFs (Feldman and Braun, 1995). For a IMF *c*(*t*), one can define its Hilbert transform ĉ(*t*) and analytic signal *z*(*t*) as shown in Equation (2) and (3), respectively.

(2) $$\hat{c}\left(t\right)=\int_{-\infty }^{+\infty }c\left(\tau \right)h\left(t-\tau \right)d\tau =\frac{1}{\pi }\int_{-\infty }^{+\infty }\frac{c\left(\tau \right)}{t-\tau }d\tau \;$$

(3) $$\begin{array}{r} z\left(t\right)=c\left(t\right)+i\times ĉ\left(t\right)=A\left(t\right)e^{i\emptyset \left(t\right)} \end{array}$$

Here, ∅(*t*) is the instantaneous phase (IP) of *c*(*t*). The instantaneous frequency *f*(*t*) is the derivation of IP and *t* shown in Equation (4):

(4) $$\begin{array}{r} f\left(t\right)=\frac{1}{2\pi }\frac{d\emptyset \left(t\right)}{dt} \end{array}$$

### The computation of SWA based on EEMD

Both FFT and EEMD were used for the decomposition of the EEG signal into its constituent frequency components. Once the original EEG signal *x*(*t*) was decomposed to *n* number of IMFs (denoted as *c*_*i*_) and a residue based on EEMD, a different way to measure SWA (denoted as *EEMD-SWA*) could be proposed, as shown in Equation (5).

(5) $$\begin{array}{r} EEMD-SWA=\sum_{i=k}^{l}std\left(c_{i}\right)/\sum_{i=1}^{n}std\left(c_{i}\right) \end{array}$$

In Equation (5), function *std*(*c*_*i*_) refers to the standard derivation of IMF *c*_*i*_, and the parameters *k* and *l* are determined by the orders of the IMFs that fall into the frequency range of SWA, according to the instantaneous frequency range of each IMF. Thus, *E1EMD-SWA* actually reflects the relative power of slow waves in the signal.

### Framework of the current research

**Figure 1:**
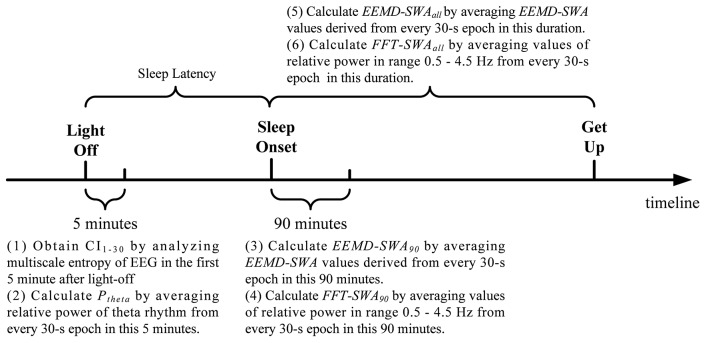
Schematic diagram of the timeline regarding EEG analysis.

In this study, EEG signals from derivation C3/A2 was imported into MATLAB for offline analysis. For each subject, two key time points were identified, light-off and sleep onset. Figure 1 briefly shows the analysis protocol. The first 5 min EEG immediately after light-out was analyzed by MSE.

On the other hand, SWA was calculated at the beginning of sleep episodes (90 min after sleep onset) and over the whole night's sleep after sleep onset, respectively. EEMD were applied to each 30 s EEG epoch in each time scope. As each 30 s EEG epoch is a time series with 3750 data points, the values of *EEMD-SWA* for all the epochs in each duration were calculated and averaged, resulting in two metrics, denoted as *EEMD-SWA*_90_ and *EEMD-SWA*_all_, for each participant. For comparison, the traditional FFT-based evaluation of SWA was applied in a similar way and resulted in another two metrics, denoted as *FFT-SWA*_90_ and *FFT-SWA*_*all*_, respectively. In the calculation of *FFT-SWA*_90_ and *FFT-SWA*_*all*_, power spectral density was estimated via the period-gram procedure with direct current filtering and Hamming windowing. SWA for each 30 s epoch was calculated by taking the power in the 0.5–4.5 Hz range as a percentage of the total signal power in the frequency range (0.5–62.5 Hz). See the Supplementary Material for the MATLAB code for *FFT-SWA*.

Then, the correlations between the proposed complexity index CI_1−30_ and sleep latency, *EEMD-SWA*_90_, *EEMD-SWA*_*all*_, *FFT-SWA*_90_, and *FFT-SWA*_*all*_, were evaluated to investigate the associations between EEG complexity and proposed measures during the wake-sleep transition and overall sleep. When sleep pressure accumulates, slow brain waves gradually become significant or dominant, which may be in line with a decline in EEG complexity. Inspired by this hypothesis, we investigated the association between brain wave complexity and sleep pressure during pre-sleep wakefulness. Theta activity (4–8 Hz), which is generally considered as a marker for the build-up of sleep pressure (Fattinger et al., 2017), was thus measured by averaging the FFT-based relative power (percentage of the power in the frequency range 0.5–62.5 Hz, denoted as *P*_theta_ in this study) for all the 30 s EEG epochs in the first 5 min after light-off, the same time course as that used for the MSE analysis.

We further analyzed whether early SWA in sleep can be predicted by the pre-sleep EEG complexity using median split subgrouping, where we divided the subjects by ranked SWA into top 50% vs. bottom 50% groups according to *EEMD-SWA*_90_ or *FFT-SWA*_90_. Such a strategy of median split has an enormous popularity in consumer research, psychology, and numerous other fields (Iacobucci et al., 2015a,b). For instance, Nordström et al. investigated whether age is a suicide risk factor for each sex by median split (Nordström et al., 2010). In addition to that, the subgrouping in the current study provided us an intuitive way to investigate the statistical difference in the values of sample entropy between the two groups over multiple time scales, making a meaningful complementation to CI_1−30_ used in regressions.

### Statistical analyses

SPSS version 19.0 (IBM SPSS Statistics, NY, United States) and MATLAB (MathWorks R2014a, Inc., Natick, MA, United States) were used for the statistical analyses. The demographics and sleep variables derived from visual scoring were reported as mean with standard deviation if data were normally distributed, and as median with lower and upper quartiles otherwise. Comparisons of the demographics and sleep variables between subgroups from the median split were assessed by chi-square test for categorical variables and unpaired *t*-test or two-sided Wilcoxon rank sum test for continuous ones. A *p* < 0.05 was considered statistically significant.

Since sleep may vary with age and sex, and high body mass index (BMI) is a strong risk factor for sleep disorders (Hou et al., 2016), these three variables were included as covariates in the statistical test for correlation between pre-sleep EEG complexity and sleep latency, as well as subsequent sleep quality. General linear models (GLM) were thus employed for the statistical analyses in SPSS 19.0 between CI_1−30_ and sleep latency, *EEMD-SWA*_90_, *EEMD-SWA*_all_, *FFT-SWA*_90_, *FFT-SWA*_all_ and *P*_*theta*_, controlling age, gender, and BMI.

For each scale in the MSE analysis, the statistical difference in the sample entropy between the *EEMD-SWA*_90_ top 50% and bottom 50% groups, as well as between the *FFT-SWA*_90_ top 50% and bottom 50% groups, were investigated by covariance analysis, controlling age, gender, and BMI. The false discovery rate (FDR) procedure was included for multiple testing.

## Results

### Modes analyses from EEMD

**Figure 2:**
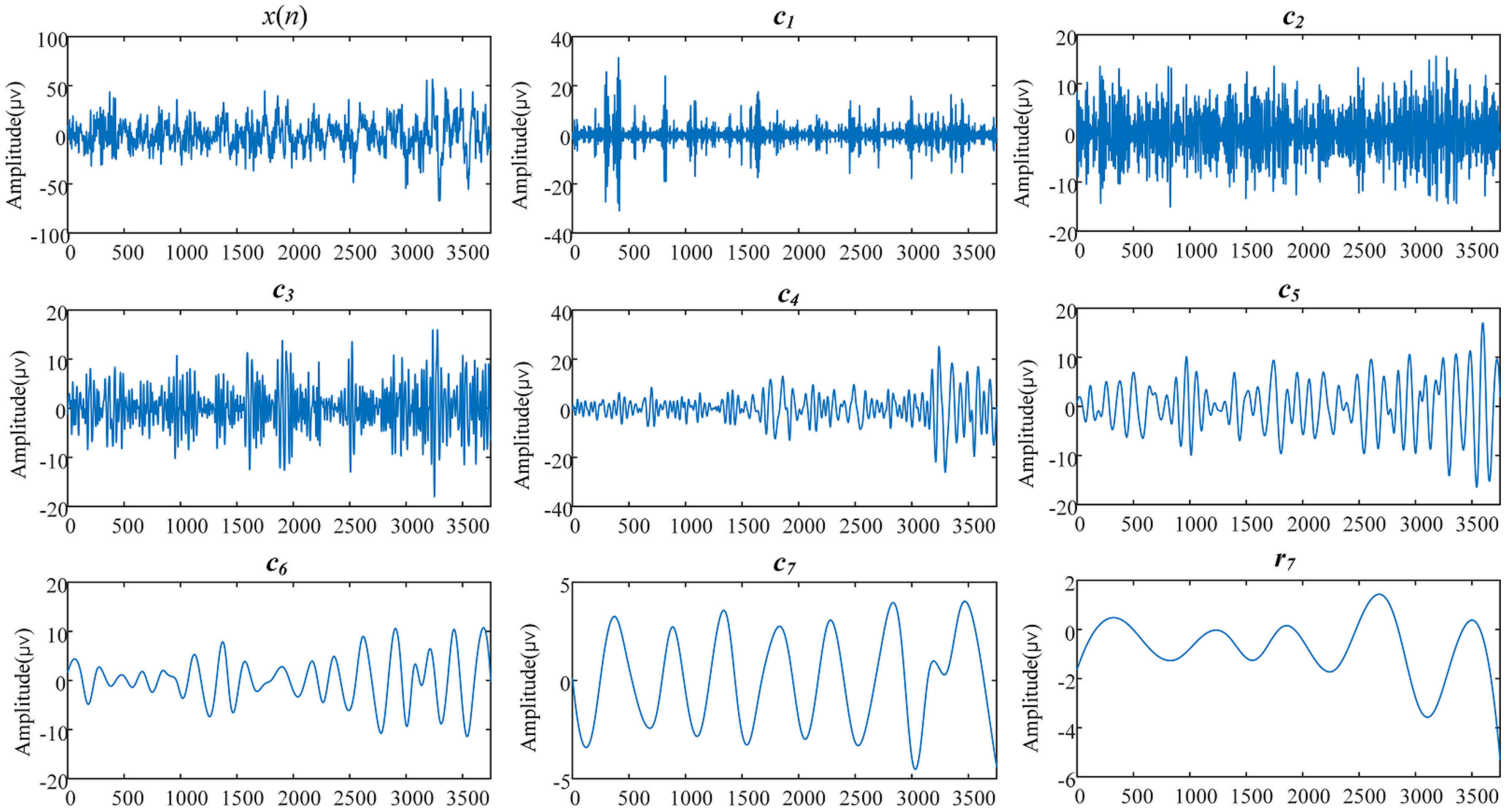
The EEMD results of a 30 s EEG signal derived from a 69-years-old woman (subject ID in SHHS-1 is 200301). *x*(*n*) is the original EEG signal, *c*_*i*_(*i* = 1,2,…,7) are the IMFs, and *r*_7_ is the residual part of data *x*(*n*) after 7 IMFs were extracted.

In practice, there are three parameters that should be determined for the application of EEMD: the ratio of the standard deviation of the noise to the target data (denoted by ε), the number of prescribed IMFs (denoted by NI), and the number of ensemble members (denoted by NE). In this study, ε was set to 0.1, NI to 7, and NE to 200. Figure 2 shows a typical result of EEMD on a 30 s EEG epoch derived from a subject in the current study.

Further estimation of the frequency range of IMFs was performed on all epochs in the current study according to Equations (2–4). For each IMF signal, Table 1 lists its frequency range, which uses two quartiles, the 2.5 and 97.5 centiles, and leaves 5% of normal outside the “normal range.” As shown in Table 1 and Figure 2, the IMF1 decomposition was mainly associated with the brain beta rhythm (13–30 Hz), while IMF2 decomposition was associated with the alpha rhythm (8–13 Hz), IMF3 decomposition with the theta rhythm (4–8 Hz), and IMF4, IMF5, IMF6, and IMF7 decompositions with the delta rhythm (< 4 Hz). Therefore, in this study, we set the values of *k, l, n* in Equation (5) to be 4, 7, and 7, respectively.

**Table 1:** Frequency range in seven IMFs of EEMD.

**Signal**	**Frequency range (Hz)**	**Associated brain rhythm**
IMF1	25.2 [20.2–38.1]	beta
IMF2	12.1 [10.3–16.9]	alpha
IMF3	6.3 [5.5–8.8]	theta
IMF4	3.5 [2.8–4.9]	delta
IMF5	2.1 [1.3–3.5]	delta
IMF6	1.5 [0.6–3.5]	delta
IMF7	1.1 [0.3–3.9]	delta

*The frequency range is expressed as median [the 2.5 centile - the 97.5 centile]*.

### Demographics and sleep variables derived from visual scoring

As shown in Table 2, the sample had a median age of 57 years and mean BMI of 25.6 kg/m^2^. A majority of the participants were identified as being of normal weight (BMI: 18.5–25 kg/m^2^) or overweight (BMI: 25–30 kg/m^2^). The standard PSG scoring results revealed that the mean TST was 371 min, while the percentages of scored sleep stages N1, N2, N3, and REM sleep were ~ 4, 55, 20, and 21%, respectively. These results are consistent with the general sleep architecture in adults (Berry, 2012).

**Table 2:** Demographics and sleep variables derived from visual scoring.

	**All**	***EEMD-SWA**_**90**_*		***FFT-SWA90***	
	***N* = 103**	**top 50% *N* = 52**	**bottom 50% *N* = 51**	**top 50% *N* = 52**	**bottom 50% *N* = 51**
Gender	19M/84F	6M/46F	13M/38F	7M/45F	12M/39F
Age (years)	57.3 ± 11.4	58.8 ± 11.9	55.8 ± 10.8	57.6 ± 11.9	57.0 ± 11.0
Body mass index (kg/m^2^)	25.6 ± 4.1	25.8 ± 4.3	25.3 ± 4.0	25.6 ± 4.3	25.6 ± 4.0
Total sleep time (min)	370.5 ± 58.6	379.6 ± 58.2	361.1 ± 58.0	380.8 ± 61.2	360.0 ± 54.3
Wake after sleep onset (min)	[20.5,62.5]	[19.0,55.8]	[22.9,69.9]	[18.3,52.0]	[25.8,67.9]*
Stage N1 sleep (%)	[2.6,5.2]	[2.6,4.7]	[2.5,5.3]	[2.4,4.2]	[2.7,5.7]*
Stage N2 sleep (%)	54.9 ± 10.9	53.1 ± 9.3	56.8 ± 12.2	51.8 ± 9.0	58.2 ± 11.9*
Stage N3 sleep (%)	20.3 ± 11.8	21.4 ± 11.3	19.1 ± 12.2	23.3 ± 11.2	17.2 ± 11.6*
REM sleep (%)	[16.7,24.6]	[18.8,25.4]	19.4 ± 5.3*	21.2 ± 4.9	19.8 ± 5.9
Sleep latency (min)	[10.0,34.0]	[8.3,29.3]	[10.5,34.4]	[8.0,29.3]	[10.6,34.4]
Slow wave activity (%)		0.744 ± 0.046	0.635 ± 0.086*	0.752 ± 0.037	0.626 ± 0.078*

Descriptive statistics were reported as mean ± standard deviation if data are normally distributed and as median [lower quartile, upper quartile] otherwise. The symbol ‘*' indicates significant difference (p < 0.05, unpaired t-test in the case of normally distributed data or two-sided Wilcoxon rank sum test in other case) between values of the bottom 50% and the corresponding top 50% group.

The demographic characteristics were balanced between the top and bottom 50% groups, with no significant differences regarding age, gender, or BMI. Sleep variables for the *EEMD-SWA*_90_ top 50% and bottom 50%, as well as for the *FFT-SWA*_90_ top 50% and bottom 50%, are also shown in Table 2. Between the groups divided by *EEMD-SWA*_90_, significant differences were found between REM sleep and SWA. However, for the *FFT-SWA*_90_ top 50% and bottom 50% groups, in addition to the quantified SWA, WASO, N1, N2, and N3 also showed significant between-group differences. For studied measures (Table 2), no significant differences were found between the two partitions when the same grouping rules (top 50% vs. bottom 50%) were applied.

### Association between EEG complexity and sleep measures

As shown in Table 3, GLM revealed a significant positive correlation between CI_1−30_ and sleep latency (*r* = 0.328, *p* = 0.001), indicating that higher complexity level was moderately associated with prolonged sleep latency.

**Table 3:** Correlations between pre-sleep EEG complexity or demographics and sleep measures in GLM models.

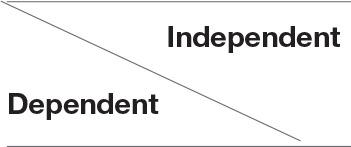	**CI**_**1−30**_		**Age**		**Gender**		**BMI**	
	**R**	**P**	**R**	**P**	**R**	**P**	**R**	**P**
Sleep Latency	0.328	0.001	0.074	0.434	0.080	0.398	−0.059	0.541
*EEMD-SWA*_90_	−0.190	0.060	0.024	0.805	0.065	0.513	0.009	0.931
*FFT-SWA*_90_	−0.165	0.103	−0.035	0.729	0.029	0.771	−0.093	0.358
*EEMD-SWA*_all_	−0.017	0.863	−0.058	0.561	0.009	0.930	0.202	0.046
*FFT-SWA*_all_	0.063	0.529	−0.123	0.220	−0.051	0.607	0.125	0.217
*P_*theta*_*	0.373	0.0001	−0.021	0.822	0.071	0.449	0.095	0.316

*R, correlation coefficient; P, probability*.

In the regression model, where we adjusted for age, gender, and BMI, *EEMD-SWA*_90_ showed a statistical trend for a weak correlation with CI_1−30_ (*r* = −0.190, *p* = 0.06), whereas no association was found between *FFT-SWA*_90_ and CI_1−30_. However, for the whole-night data, no correlation was found between CI_1−30_ and *EEMD-SWA*_*all*_ or *FFT-SWA*_*all*_. Furthermore, the GLM model, controlling age, gender, and BMI, revealed a significant positive correlation (*r* = 0.373, *p* = 0.0001) between *P*_*theta*_ and CI_1−30_.

**Figure 3:**
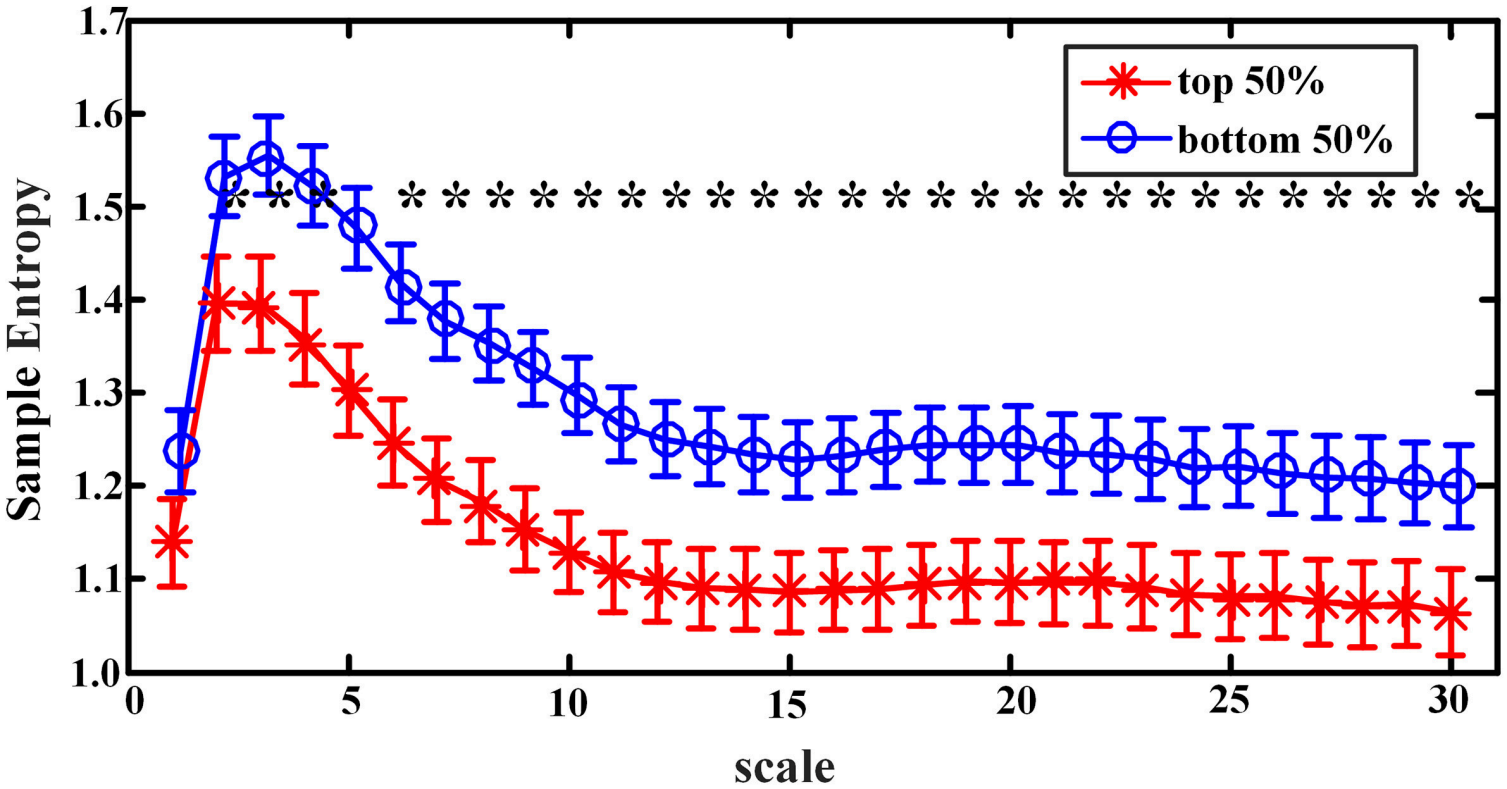
Complexity indices (sample entropy, mean ± SD) on scale 1–30 in groups classified by the rank of *EEMD-SWA*_90_ (top 50% vs. bottom 50%). The symbol ‘*' indicates significant difference between groups (*p* < 0.05, covariance analysis, controlling the age, gender, and BMI). After the procedure of FDR, the significant differences all remained.

**Figure 4:**
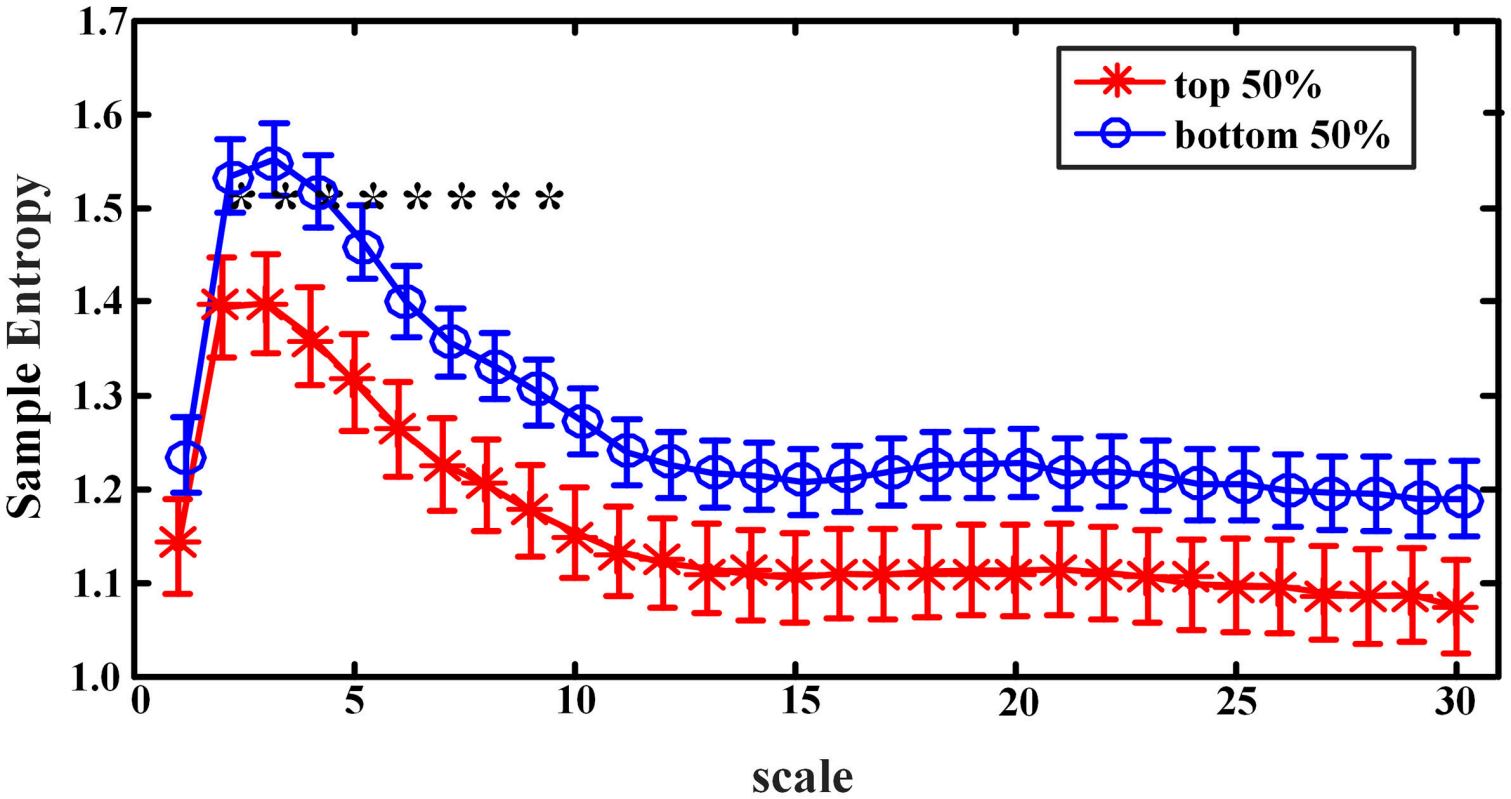
Complexity indices (sample entropy, mean ± standard deviation) on scale 1–30 in groups classified by the rank of *FFT-SWA*_90_ (top 50% vs. bottom 50%). The symbol ‘*' indicates significant difference between groups (*p* < 0.05, covariance analysis, controlling the age, gender and BMI). However, after the procedure of FDR, none of the significant differences remained.

When sample entropies on scales 1–30 were compared between the *EEMD-SWA*_90_ top 50% and bottom 50% groups, significant differences (controlled by the FDR procedure) were found on all the chosen scales, except that of scale 1. As presented in Figure 3, the group with the higher *EEMD-SWA*_90_ in sleep showed a reduced complexity of pre-sleep brain dynamics. When the *FFT-SWA*_90_ top 50% and bottom 50% groups were compared, as shown in Figure 4, a similar negative correlation was observed, but significant differences (*p* < 0.05) between the two groups were found only on the short time scales (scales 2–9). However, after FDR controlling, no significant differences remained for any of the 30 scales, indicating that the EEMD-based method might provide a more robust way to measure SWA.

## Discussion

In this study, we examined the associations among wake EEG complexity, sleep latency, and the subsequent SWA quantities during early sleep and over the entire night. Our results revealed a positive correlation between the complexity during the 5 min wakeful EEG and sleep latency. Such complexity also showed a statistical trend for a weak and negative correlation with EEMD-SWA at the beginning of sleep (90 min after sleep onset). Our results suggest that the lower complexity of brain waves in the pre-sleep state may facilitate sleep initiation and reduce sleep latency.

As mentioned in the Introduction, high mental load and urgency to fall asleep increase sleep onset latency (Ansfield et al., 1996) and may lead to non-restorative and unsatisfying sleep, especially in patients with mental disorders or with sleep-onset insomnia, that is, difficulty in sleep initiation. Existing evidence suggests that the manipulation of pre-sleep cognitive activity can lead to changes in sleep onset latency (Ansfield et al., 1996; Nelson and Harvey, 2003; Wuyts et al., 2012). Therefore, future studies are encouraged to examine the complexity before sleep among such populations and to investigate whether interventions that reduce brain wave complexity can assist in promoting restful sleep and improved sleep quality by increasing SWA. In addition, as previously introduced, when a system is under stress or relatively relaxed, levels of complexity can be an appropriate indicator (Costa et al., 2002a, 2005; Goldberger et al., 2002). In recent years, some studies used complexity measures to quantify or evaluate the relaxation states (Aftanas and Golocheikine, 2002; Natarajan et al., 2004). Therefore, we can assume that complexity measures may be useful to examine relaxation interventions such as meditation, slow-paced breathing, music therapy, and cognitive therapy. Effective approaches might be expected to reduce pre-sleep brain complexity and therefore improve sleep quality.

Although in adults, a pronounced increase of theta activity has often been found during the course of sleep deprivation (Aeschbach et al., 1997, 1999; Finelli et al., 2000; Strijkstra et al., 2003; Fattinger et al., 2017), we observed a counterintuitive link between EEG complexity and theta activity during wakefulness in the studied population with normal sleep rather than sleep deprivation. Owing to the counteraction of the circadian process, a linear function with a superimposed 24 h sine wave is generally fitted to theta power and time awake (Åkerstedt and Gillberg, 1990; Finelli et al., 2000). In this way, theta activity may not increase within a session until extreme sleepiness is encountered (Åkerstedt and Gillberg, 1990; Finelli et al., 2000). Aeschbach et al. investigated the influence of the circadian pacemaker and of the duration of time awake on EEG (Aeschbach et al., 1999). They found that the theta rhythm was exhibited a minimum of 1 h after the onset of melatonin secretion (the clock time of melatonin onset in Aeschbach's study ranged from 22:05 to 22:43), indicating evident dissociation, as the circadian maximum of theta activity appeared to be delayed with respect to the maximum of sleep propensity. In our study, 72 out of 103 participants reported their clock time for light-off in the range of 22:05–23:43, however, it is still lack of evidence to make clear the association between sleep pressure and EEG complexity before nocturnal sleep in normal populations. The association between complexity/MSE and sleep-wake regulation is therefore not straightforward and deserves further investigations.

We also analyzed SWA over the entire night for each subject. However, those whole-night results showed significantly weaker correlations with wake complexity compared with SWA_90_. As it is well-recognized that SWA exhibits a global declining trend over the course of the night and its level in the first non-REM episode increases as a function of prior waking (Achermann et al., 1993), which may indicate the reason why the observed association only present between pre-sleep EEG complexity and SWA in early sleep rather than the whole night. Nonetheless, it would be worth investigating the dynamic changes in pre-sleep complexity and SWA through the entire night, and examining the potential causal relationship in future work. As mentioned above, most of the previous studies of EEG complexity focused on analyses of entropy for each sleep stage, showing a trend of decreasing complexity as sleep becomes deeper (Ma et al., 2018). Non-linear features, including complexity indices, may provide assistance in automatic sleep classification, but, more importantly, studies are encouraged to break the boundaries of limitation to expand the application of non-linear approaches, so that we can better understand the sleep dynamics (Ma et al., 2018). A further step would be to study the complexity differences between normal and pathological conditions, and investigate whether abnormal sleep can be predicted by the first few minutes of pre-sleep EEG recording while a subject is still awake. It would also be of value to determine the potential mechanisms of pre-sleep EEG complexity and sleep-wake transitions and overall sleep quality. Again, we encourage broad applications of such complexity-based approaches in future studies.

Regarding the methodology, the groups defined by FFT-SWA_90_ showed significant differences regarding manually scored sleep parameters (e.g., WASO, N1, N2, N3), while the differences were not significant when groups were defined by EEMD-SWA_90_. One possible reason is that both manual scoring and FFT spectral analysis are based on the wave morphology and feature waves. However, for revealing the underlying association between wake complexity and quantified sleep SWA, the proposed EEMD-based method had better performance than traditional FFT-based spectral analysis. GLM revealed a weak correlation between pre-sleep EEG complexity and EEMD-SWA during the first 90 min of sleep. Furthermore, when we split the participants into two groups according to the values of *EEMD-SWA*_90_, the sample entropies at almost all time scales exhibited significant differences between the two groups, suggesting that waking EEG complexity can distinguish between lower and higher groups of SWA after sleep onset quantified by the EEMD approach. However, when SWA was measured using a traditional way such as FFT, any association between waking complexity and sleep SWA was insignificant. Owing to the non-linear and non-stationary characteristics of EEG signals during sleep, there would be a drifting of the frequency ranges of the EEG components for each individual. In this situation, as demonstrated in the current study, compared with the frequency band fixed method, a data adaptive approach such as EEMD to measure SWA would be more sensitive to the underlying dynamics of sleep and better able to uncover its mechanisms.

One strength of this study was that we used an existing database and included a reasonably large number of subjects provided by the freely available database (SHHS) to test our hypothesis. Meanwhile, limitations of this study include that associations measured at the population level may not reflect associations at the individual level, and that the study is prone to confounding by other factors. In our study, 82% of the participants were female, which may limit the generalizability of our finding, given that hormonal changes during the menstrual cycle or in peri-menopausal transition may affect sleep (Mallampalli and Carter, 2014; Jehan et al., 2015). However, no such information was provided for the participants in the database. Future studies with prospective designs are strongly encouraged. In addition, the PSG data provided in SHHS-1 were collected from two scalp-placed electrodes: C3 and C4. Since each brain region is associated with different EEG feature waves, future studies are encouraged to perform such complexity and SWA analyses using multiple EEG montages.

In conclusion, lower complexity before sleep onset is associated with a decline of sleep latency and higher SWA after sleep onset, suggesting that reduced complexity of brain waves may improve sleep quality. The application of complexity measures is important to extend our knowledge of sleep. Future studies are encouraged to explore the complexity before sleep among subjects with sleep disorders, to determine its relationship with the efficacy of interventional approaches or for developing screening tools for use with short-term pre-sleep EEG. Although the current study elucidated the association between wakeful brain complexity and nocturnal sleep quality in the healthy population, future investigations should focus on patients with sleep disorders. We would like to encourage interdisciplinary efforts to address this research question.

## Ethics statement

The study protocol of SHHS was approved by the institutional review board of each participating center, and each participant signed informed consent. All methods were carried out in accordance with relevant guidelines and regulations. The current study only analyzed de-identified data from the SHHS database, and did not involve a research protocol requiring approval by the relevant institutional review board or ethics committee.

## Author contributions

FH, C-KP, CW, and YM designed this study. FH, AY and ZY analyzed data. FH and YM wrote the article.

### Conflict of interest statement

The authors declare that the research was conducted in the absence of any commercial or financial relationships that could be construed as a potential conflict of interest. The reviewer IK and handling Editor declared their shared affiliation.
