# Supplementary material for: Complexity of Wake Electroencephalography Correlates With Slow Wave Activity After Sleep Onset
Source: Front Neurosci. 2018 Nov 13;12:809. doi: 10.3389/fnins.2018.00809 (PMC6243118; doi:10.3389/fnins.2018.00809)
Supplement: Supplementary file 1 [file Table_1.DOCX]

Supplementary Material

Complexity of Wake Electroencephalography Correlates with Slow Wave Activity after Sleep Onset

Fengzhen Hou^*^, Zhinan Yu, Chung-Kang Peng，Albert Yang, Chunyong Wu，Yan Ma

*** Correspondence:** Corresponding Author: [houfz@cpu.edu.cn](mailto:houfz@cpu.edu.cn), cywu@cpu.edu.cn

# Supplementary Data

The identifiers of the included subjects, which were created by the National Sleep Research Resource (NSRR) team to more easily match with file downloads, were shown as following.

| 200170; | 200437; | 201210; | 202508; | 203027; | 203389; | 204171; | 204702; | 204836; | 204927; |
| --- | --- | --- | --- | --- | --- | --- | --- | --- | --- |
| 200172; | 200500; | 201250; | 202545; | 203030; | 203392; | 204175; | 204734; | 204846; | 204961; |
| 200223; | 200691; | 201371; | 202715; | 203044; | 203514; | 204230; | 204737; | 204858; | 205270; |
| 200224; | 200710; | 201415; | 202723; | 203058; | 203681; | 204259; | 204749; | 204865; | 205285; |
| 200301; | 200718; | 201423; | 202729; | 203063; | 203750; | 204298; | 204764; | 204871; | 205497; |
| 200319; | 200852; | 201452; | 202735; | 203096; | 203782; | 204376; | 204766; | 204887; | 205509; |
| 200362; | 200902; | 201461; | 202857; | 203137; | 204027; | 204413; | 204777; | 204888; | 205619; |
| 200393; | 200903; | 201688; | 202917; | 203230; | 204061; | 204415; | 204803; | 204894; | 205679; |
| 200404; | 200915; | 201970; | 202963; | 203377; | 204145; | 204441; | 204822; | 204910; | 205692; |
| 200435; | 201120; | 202261; | 202983; | 203387; | 204166; | 204614; | 204835; | 204911; | 205702; |
| 205710; | 205723; | 205785 |  |  |  |  |  |  |  |

# MATLAB code for Multiple Sample Entropy (MSE) analysis

function SampEnVal = SampEn(data, m, r)

data = data(:)';

N = length(data);

Nkx1 = 0;

Nkx2 = 0;

for k = N - m:-1:1

x1(k, :) = data(k:k + m - 1);

x2(k, :) = data(k:k + m);

end

for k = N - m:-1:1

x1temprow = x1(k, :);

x1temp = ones(N - m, 1)*x1temprow;

dx1 = max(abs(x1temp - x1), [], 2)';

Nkx1 = Nkx1 + (sum(dx1 < r) - 1)/(N - m - 1);

x2temprow = x2(k, :);

x2temp = ones(N - m, 1)*x2temprow;

dx2 = max(abs(x2temp - x2), [], 2)';

Nkx2 = Nkx2 + (sum(dx2 < r) - 1)/(N - m - 1);

end

Bmx1 = Nkx1./(N - m);

Bmx2 = Nkx2./(N - m);

SampEnVal = -log(Bmx2./Bmx1);

end

function MSE = Multiscale_SampEn( X,m,r,Scale )

MSE=[];

for j=1:Scale

Xs = Multi(X,j);

MSE(j) = SampEn(Xs',m,r);

end

# MATLAB code for the calculation of EEG band power

function Results=RelativePower(x,Fs,N)

x=x-mean(x);

freq=[4 8; 0.5 4.5];

nbands=length(freq);

[pxx,~]=periodogram(x, hamming(N), N, Fs);

Ptotal=sum(pxx(round(0.5*N/Fs)+1:round(62.5*N/Fs)-1))*Fs/N;

for i=1:nbands

ns=round(freq(i,1)*N/Fs);

ne=round(freq(i,2)*N/Fs)-1;

Pband(i)=sum(pxx(ns:ne))*Fs/N;

end

Results=Pband/Ptotal;
